# Efficacy of repetitive transcranial magnetic stimulation combined with visual scanning treatment on cognitive and behavioral symptoms of left hemispatial neglect in right hemispheric stroke patients: study protocol for a randomized controlled trial

**DOI:** 10.1186/s13063-020-04943-6

**Published:** 2021-01-06

**Authors:** Francesco Di Gregorio, Fabio La Porta, Emanuela Casanova, Elisabetta Magni, Roberta Bonora, Maria Grazia Ercolino, Valeria Petrone, Maria Rosaria Leo, Roberto Piperno

**Affiliations:** 1 Azienda Unità Sanitaria Locale, UOC di Medicina Riabilitativa e Neuroriabilitazione, Bologna, Italy; 2 IRCCS Istituto delle Scienze Neurologiche di Bologna, UO di Medicina Riabilitativa e Neuroriabilitazione, Casa dei Risvegli Luca de Nigris, Via Giulio Gaist, 6, 40139 Bologna, Italy; 3 Villa Bellombra rehabilitation Hospital, Bologna, Italy

**Keywords:** Stroke rehabilitation methods, Transcranial magnetic stimulation, Brain pathology, Brain physiology, Functional laterality, Randomized controlled trial

## Abstract

**Background:**

Left hemispatial neglect (LHN) is a neuropsychological syndrome often associated with right hemispheric stroke. Patients with LHN have difficulties in attending, responding, and consciously representing the right side of space. Various rehabilitation protocols have been proposed to reduce clinical symptoms related to LHN, using cognitive treatments, or on non-invasive brain stimulation. However, evidence of their benefit is still lacking; in particular, only a few studies focused on the efficacy of combining different approaches in the same patient.

**Methods:**

In the present study, we present the SMART ATLAS trial (**S**timolazione **MA**gnetica **R**ipetitiva **T**ranscranica nell’**AT**tenzione **LA**teralizzata dopo **S**troke), a multicenter, randomized, controlled trial with pre-test (baseline), post-test, and 12 weeks follow-up assessments based on a novel rehabilitation protocol based on the combination of brain stimulation and standard cognitive treatment. In particular, we will compare the efficacy of inhibitory repetitive-transcranial magnetic stimulation (r-TMS), applied over the left intact parietal cortex of LHN patients, followed by visual scanning treatment, in comparison with a placebo stimulation (SHAM control) followed by the same visual scanning treatment, on visuospatial symptoms and neurophysiological parameters of LHN in a population of stroke patients.

**Discussion:**

Our trial results may provide scientific evidence of a new, relatively low-cost rehabilitation protocol for the treatment of LHN.

**Trial registration:**

ClinicalTrials.gov NCT04080999. Registered on September 2019.

## Background

Left hemispatial neglect (LHN) is a disabling neuropsychological syndrome, which occurs in about 30% of persons with stroke [1]. LHN is a well-known predictor of poor long-term functional outcome [2], as it may be associated with more extended in-hospital stay, long term limitations in activities of daily living, and an increased risk of falling [3]. About 90% of persons with LHN have suffered a right hemispheric stroke [1]. Patients with LHN present deficits in perceiving, responding, consciously representing, and orienting attention toward the contralesional hemifield, usually the left hemifield [4, 5]. Consequently, this specific condition leads to a lack of awareness for objects, persons, and even for their own body parts in the left hemifield. Furthermore, LHN can be associated with a deficit in other clinical domains. Indeed, motor deficits involving the body’s contralateral side (i.e., left hemiplegia) are often present. This clinical picture can be aggravated by a lack of awareness for cognitive and motor deficits (i.e., anosognosia), particularly influencing rehabilitation and daily living activities [6]. Thus, the complexity of symptoms in LHN requires broad assessments and specific rehabilitation protocols.

Clinically, LHN is investigated with paper and pencil neuropsychological tests (e.g., lines bisection, letter, or star cancelation), which assess the ability to search for stimuli in space. Recently, however, neurophysiological diagnostic procedures were introduced for the functional evaluation of LHN. Specific paradigms indeed use visual evoked potentials (VEPs) [7, 8] to assess biases in the processing of lateralized (i.e., right or left) presented stimuli. These studies evidenced a correlation between visual-spatial attention deficits in LHN and a reduction in amplitude and later latencies of the N100 component for stimuli presented in the left neglected hemifield. N100 anomalies are observed both at the left and right parietal cortices and on frontoparietal areas. These results suggest that functional impairments in LHN involve both hemispheres and the cerebral network involved in spatial attention orientation [1, 9]. Indeed, influential theories sustained the idea that LHN is related to a dysfunction in the neural network subtending attentive spatial processing. In particular, several studies report an imbalance in interhemispheric activity due to hyperactivity of the left hemisphere in LHN patients [1, 10–12].

In the rehabilitation of LHN, it is possible to distinguish protocols based on cognitive treatments and non-invasive brain stimulation, such as repetitive transcranial magnetic stimulation (r-TMS) [13, 14]. Conventional cognitive treatments (CCT) of LHN involve different types of exercises aimed at reducing attentional bias for the ipsilesional space (e.g., prism adaptation) and promoting contralesional space awareness (e.g., visual scanning training) [15]. Although several studies demonstrated the efficacy of CCTs on improving short and medium-term LHN symptoms, the debate is still open about these treatments’ long-term efficacy [16]. Thus, over the past 20 years, the interest in rehabilitation approaches based on non-invasive brain stimulation has increased. In particular, transcranial magnetic stimulation (TMS) is a non-invasive method to modulate the cerebral cortex’s excitability. For instance, Brighina et al. (2003) [17] applied low-frequency (1 Hz) inhibitory repetitive TMS (r-TMS) in the healthy contralesional areas. The r-TMS treatment improved the clinical symptoms of LHN after ten treatment sessions. Improvements were stable even 15 days after treatment. This study provided encouraging initial evidence on the efficacy of interventions based on TMS. Indeed, several studies also investigated the combined and additive effects of various rehabilitation techniques on the same patient (i.e., TMS and CCT) [18–20] to potentiate the intervention.

However, methodological limitations emerged from these studies, such as reduced sample sizes, the lack of blindness of assessors, the lack of a general assessment of activities of daily living, and long-term follow-up. Thus, it is still necessary to understand whether the combination of different therapeutic approaches can have a clinically significant additive effect in reducing the severity of symptoms of LHN. Furthermore, combined protocols need to be studied in terms of their neurophysiological correlates to quantify better their effects over the interhemispheric imbalance and in terms of long-lasting clinical results.

This study’s central hypothesis is that, as observed in previous studies for right hemispheric strokes, clinical symptoms of LHN are correlated to an imbalance in interhemispheric activity due to hyperactivity of the left hemisphere [1, 10–12]. Inhibitory r-TMS on the intact left parietal cortex reduces the left hemisphere activation and can rebalance interhemispheric activity. Indeed, the most used TMS paradigms for the rehabilitation of LHN are based on the concept of interhemispheric rivalry [21, 22]. According to this model, the two hemispheres exert a reciprocal inhibition. In particular, the right and left parietal cortices are part of an interhemispheric and intrahemispheric frontal-parietal pathway that controls the orientation of attention in space [1]. Thus, the right parietal cortex’s damage causes disinhibition of the left parietal cortex and, therefore, a pathological over-activation of the latter. This left over-activation consequently inhibits the right contralateral hemisphere by increasing the healthy hemisphere’s inhibitory activity on the damaged hemisphere. TMS treatments, therefore, aims at rebalancing interhemispheric rivalry by stimulating the parietal interhemispheric pathway, specifically inhibiting the areas of the left healthy parietal cortex [5, 23]. Thus, should r-TMS administered before a cognitive treatment, it might reduce the interhemispheric imbalance. Consequently, r-TMS may increase brain activity toward the left neglected hemifield and boost the effects of cognitive training. Specifically, in the present study, we hypothesize that a combined r-TMS and cognitive training protocol could be efficient in rehabilitating clinical and cognitive symptoms of LHN, reducing interhemispheric imbalance with a positive impact on activities of daily living.

### Aims and objectives

The aims of this study are: (1) to test the central hypothesis by assessing the immediate and long-term efficacy of a combined treatment based on r-TMS and visual scanning training (a conventional cognitive protocol based on the administration of a structured series of tasks aiming at improving spatial exploration abilities [13, 24] on the cognitive and behavioral manifestation of LHN in a population of patients with right-hemisphere stroke), (2) to assess the immediate and long-term efficacy of the proposed treatment on independence in activities of daily living, and (3) to investigate the clinical responsiveness of neurophysiological correlates and indexes based on visual evoked potentials (VEPs).

Our operational objectives and outcome measures are divided into primary and secondary outcomes to reach all these aims. The primary outcome is represented by (1) a specific assessment of visual-spatial attentive functions and behavioral symptoms of LHN with the Behavioral Inattention Test (BIT) [24]. The secondary endpoints consider the impact of the protocols on other clinical and neurophysiological indexes. In particular, we aim to test (2) the degree of functional independence with the Catherine Bergegò Scale, which considers the impact of LHN symptoms during activities of daily living (ADL) [25]. Furthermore, we test mobility with Motricity Index, Trunk Control Test, and Functional Independence Measure (FIM) [2, 26] that are measures of motor impairments, attentive functions with the Test of Attentional Performance (TAP), that is a computerized attention assessment. Finally, we tested (3) neurophysiological indexes of interhemispheric imbalance based on the latencies (IHTT, interhemispheric transmission time) and amplitudes (vABI, Visuospatial Attention Bias Index) of the N100 components of the VEPs.

## Methods

### Trial design and study design

**Fig. 1:**
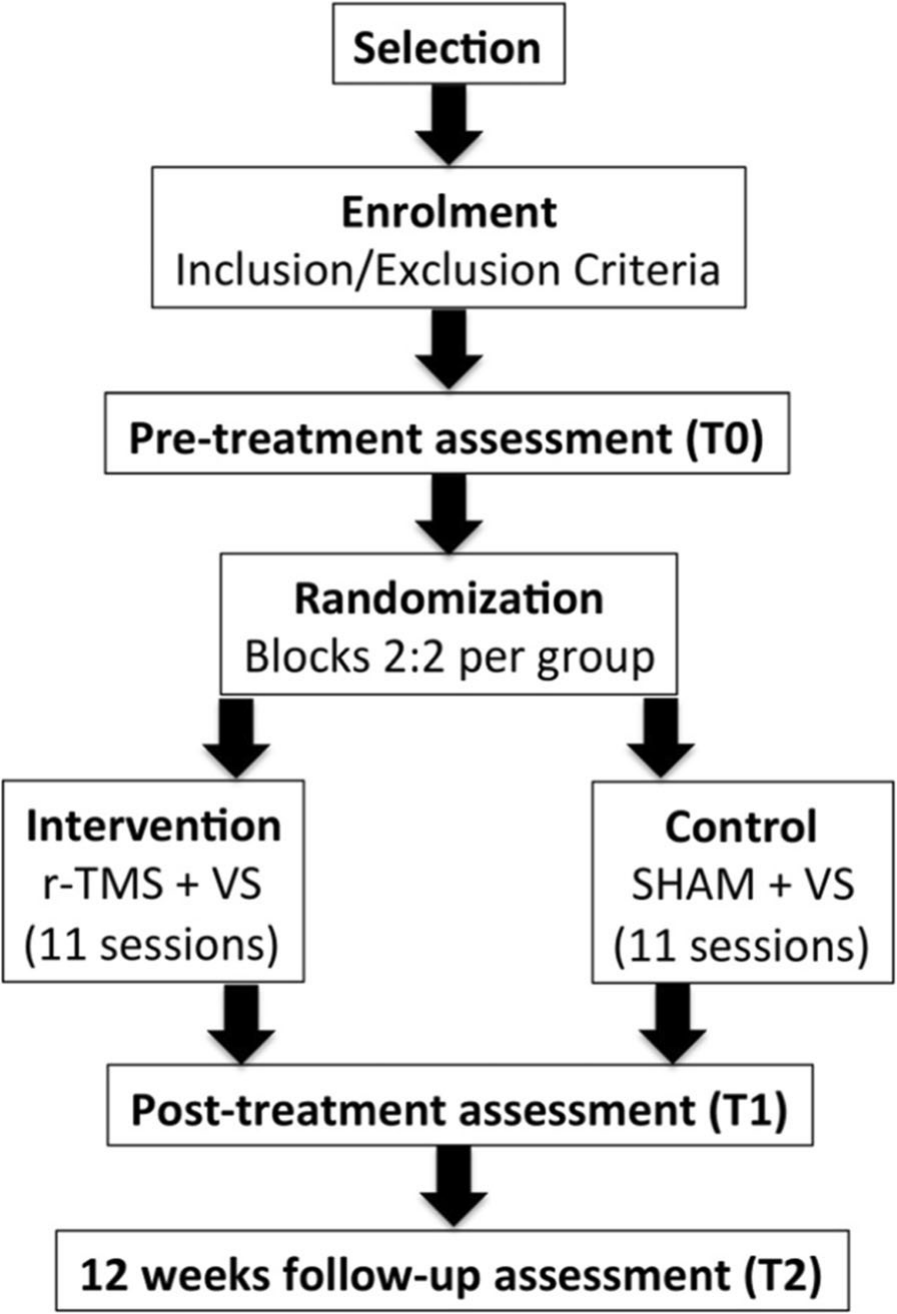
Study flowchart. r-TMS, repetitive transcranial magnetic stimulation; VS, visual scanning

We present the SMART ATLAS trial (**S**timolazione **MA**gnetica **R**ipetitiva **T**ranscranica nell’**AT**tenzione **LA**teralizzata dopo **S**troke; in English: repetitive transcranial magnetic stimulation in lateralized attention after stroke), a multicenter, randomized, controlled trial with pre-test (baseline), post-test, and 12 weeks follow-up assessments aiming at comparing the efficacy of inhibitory r-TMS, applied over the left intact parietal cortex of LHN patients, followed by visual scanning treatment (VS) [27], in comparison with a placebo stimulation (SHAM control) followed by the same visual scanning treatment, on visuospatial symptoms of LHN in a population of stroke patients. Our methodological approach provides two parallel groups (active r-TMS and SHAM placebo groups) with a 2:2 randomized allocation ratio in a superiority trial design. Figure 1 and Table 1 show the study flowchart and the study time points, respectively.

**Table 1:** Study time points

Study period							
Stages ➔	Enrolment	Pre-allocation	Allocation	Post-allocation			Follow-up
**Weeks**	W0	W0	W0	W1	W2	W3	W15
**Time points**	T_0_			T_1_			T_2_
**Eligibility Screening**	X						
**Informed Consent**	X						
**Allocation**			X				
**Treatment**				X	X		
**Assessments**		X				X	X

### Participants

Patients with LHN due to stroke will be recruited in the Neurorehabilitation Unit of the IRCCS Istituto delle Scienze Neurologiche di Bologna (coordinating center) and the Villa Bellombra rehabilitation Hospital in Bologna (Italy). Subjects will be recruited accordingly to the following eligibility criteria:

Inclusion criteria:

1. Diagnosis of ischemic stroke of the right medial cerebral artery or right intracranial hemorrhagic stroke, confirmed by encephalic CT scan or MRI;
2. Diagnosis of LHN with specific screening test (asymmetry score in the Bells test > 3);
3. Inpatient or outpatient rehabilitation setting;
4. Age between 18 and 80 years;
5. Time after stroke between 3 weeks and 12 weeks; and
6. Adequate language comprehension to give informed consent.

Exclusion criteria:

1. Medical instability at the time of enrollment, defined as the acute onset of an unexplained derangement of one or more vital parameters (temperature, blood pressure, pulse rate, respiratory rate, oxygen saturation, level of responsiveness) outside the normal range (e.g., fever, cardiac arrhythmias, respiratory insufficiency) and/or the onset of any new medical condition requiring unexpected additional diagnostic procedures and treatments (e.g., severe pain, reduction of urinary output);
2. Presence of epileptogenic alterations of the EEG and/or previous epileptic seizures;
3. Presence of intracranial implants of a metallic material;
4. Presence of devices that could be altered by r-TMS, such as pacemakers, ventriculoperitoneal derivations, or baclofen infusion pumps;
5. Absence of bone flap following decompressive craniectomy;
6. Presence of alteration in the consciousness-vigilance rhythm;
7. Cortical blindness and/or visual agnosia;
8. Previous psychiatric disorders and/or history of substance abuse;
9. Pregnancy state;
10. Severe deafness not compensated by hearing devices;
11. Severe reduction of visual acuity not compensated by optic lenses; and
12. Previous diagnosis of cognitive impairment.

Before enrollment in the study, the principal investigator (PI) will check the eligibility criteria. In particular, after verifying the eligibility criteria, the PI (or a delegate) will provide the potentially eligible person with all the information and details relative to the study in a simple language during an interview that will preferably take place in the presence of a caregiver.

### Intervention

Based on previous studies [5, 17, 23], seven sessions of r-TMS will be administered over 15 days [17]. In detail, the parameters used in each session will be:

1. International 10/20 system for the location of the target area (non-lesioned left posterior parietal cortex);
2. 60% power;
3. Frequency: 1 Hz; and
4. Ninety pulse trains with ten pulses each (total 900 stimuli) resulted in a whole stimulation period of 15 min.

The stimulation coil will be positioned tangentially on the target area. Each r-TMS session will last 15 min and be administered every other day (e.g., Monday-Wednesday-Friday, Monday-Wednesday-Friday, Monday).

Following the rTMS, the visual scanning treatment will be administered by speech and language therapists and cognitive therapists, who will administer various visual scanning tasks, increase the patient’s awareness of the LHN clinical manifestations, and teach the participant strategies to improve spatial exploration abilities [23]. In particular, three different training protocols will be used:

1. Visuospatial training;
2. Reading and copying training; and
3. Copying of line drawings on a dot matrix.

All training protocols include three increasing levels of difficulty, thus giving nine possible test-difficulty combinations. Each level of difficulty will be practiced until the subject will reach a level of accuracy of 75%. The training will be carried out in 50 min sessions for 5 days a week within 15 days [23], for a total of 11 sessions. On the days when the r-TMS is also carried out, the visual scanning protocol administration will follow the brain stimulation.

### Control

In the control group, where a SHAM placebo stimulation is implemented, the stimulation parameters will be the same, but the coil of the r-TMS will be positioned at 90° on the target area. Thus, no specific cortical modulation will be implemented (SHAM stimulation). The visual scanning protocol will be administered with the same modalities and time frame for this group, as detailed for the intervention group. All routine care is permitted for both groups during the study period.

### Outcomes

#### Primary endpoint

The Behavioral Inattention Test (BIT), a neuropsychological battery for visual and cognitive assessment of LHN in standard paper and pencil form, represents the primary outcome. The BIT consists of two subscales (cognitive and behavioral) with standardized scores, where lower ratings indicate a more severe visual-spatial impairment.

#### Clinical secondary endpoints

In this context, subtests from the TAP battery (Test of Attentional Performance) will be administered to test the attention levels of LHN patients. In particular, we will administer the following subtest from the TAP: Alertness and Visual Field/Neglect. The Catherine Bergegò Scale (CBS) assesses specifically the presence and severity of LHN in the activities of daily living (i.e., eating or reading biases). Motricity Index (MI) and Trunk Control Test (TCT) will be used to measure motor impairments, whereas the motor scale of the FIM™ will assess dependence in ADL and mobility. In this way, it will be possible to evaluate different clinical aspects related to LHS and control other improvements induced by the rehabilitation protocol.

#### Neurophysiological secondary endpoints

**Fig. 2:**
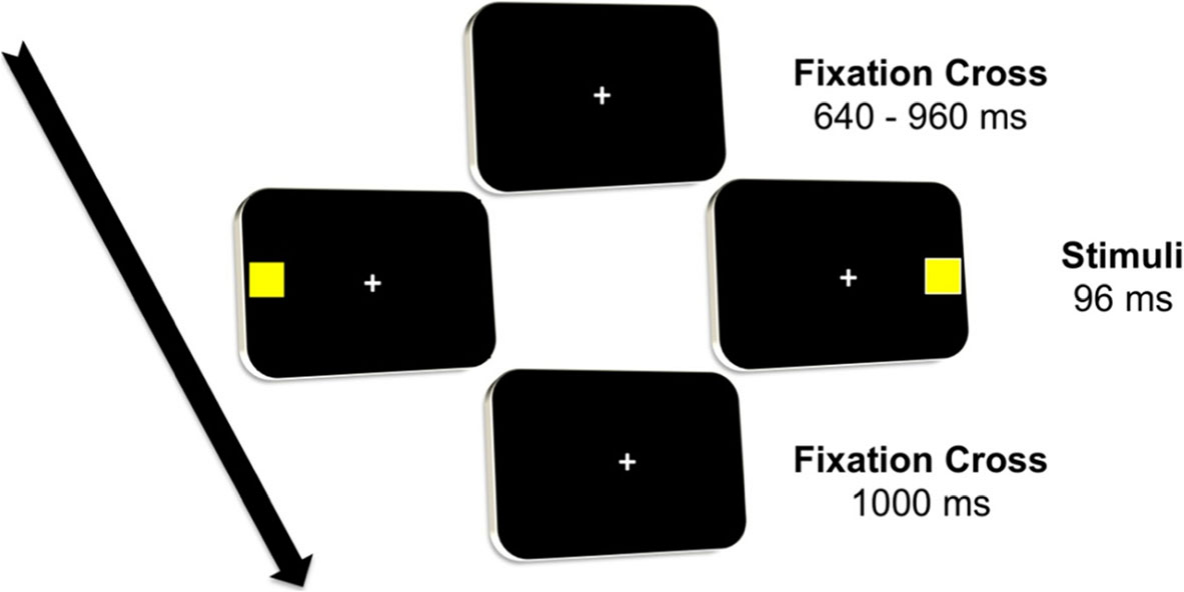
Visual detection task. Each trial starts with a central fixation cross for a jittered time between 640 and 960 milliseconds (ms), then a stimulus is presented randomly either on the left or on the right of the fixation cross for 96 ms. After stimulus presentation, a fixation cross remains for 1000 ms, and then a new trial starts

Note. The vABI will be calculated as a lateralization index [31] for the N100 amplitude. The vABI will be extracted in two steps. First, N100 for left and right presented stimuli will be calculated in the averaged activity from posterior electrodes (i.e., mean of P3 and P4) to have a signal of the two hemispheric activations after lateralized stimulus presentation. Then, for each participant, the difference in the activations for lateralized stimuli will be calculated as the difference between the resulted N100 peaks for left and right-presented stimuli (i.e., vABI = N100 amplitude for right stimuli − N100 amplitude for left stimuli). Such an index can measure interhemispheric imbalance as it reflects the difference in the activation of the two hemispheres in response to lateralized stimuli. Similarly, based on N100 peak latency, we will compute the IHTT [29, 30], an indicator of the EEG signal’s transmission times from one hemisphere to the other. In particular, we will calculate the IHTT as the difference between the latencies of the negative peak of N100 for left presented stimuli on the posterior electrodes P3 and P4 (i.e., IHTT = N100 latency on P4 - N100 latency on P3), thus constituting a single index in milliseconds of the interhemispheric transmission times specifically for left presented stimuli.

Finally, in the present study, lateralized visual processing’s neurophysiological correlates are collected to investigate the common interhemispheric imbalance in LHN [1, 10, 28]. In this study, the EEG will be recorded while patients perform a visual detection task (Fig. 2) seated at a distance of 50 cm from the computer’s screen. Patients will be prompted to fix a central fixation cross on a screen while visual stimuli are presented. Stimuli will be small yellow squares (1 cm × 1 cm) shown in a black background to detect color contrast between the stimuli and the background easily. Each trial will start with a white central fixation cross over a black background. Then, after a varying stimulus onset asynchrony between 640 and 960 ms (steps of 80 ms), a stimulus will be presented randomly on the right or the left of the fixation cross on a view distance angle 28° along the midline. Stimuli will be displayed for 96 ms; before the subsequent trial, a black background with the fixation cross will be presented for 1000 ms (Fig. 2). In general, the visual detection task consists of a passive visual task with lateralized stimuli, and participants will be prompted to keep the fixation during all tasks [7, 8]. Whenever participants lose the fixation, feedback will be provided to recover it. Overall, 256 stimuli will be presented in 4 blocks of 64 stimuli, where the overall task will have an average duration of 20 min. The EEG will be recorded from 18 Ag/AgCl-cup electrodes according to the 10/20 system referenced to the linked ear lobes. The EEG signal will be recorded from electrodes: Fz, Cz, Pz, C4, C3, P4, P3, F4, F3, Oz, O1, O2. Impedance for EEG and electrooculogram (EOG) electrodes will be kept below 10 kΩ. Off-line, the amplitude in microvolt (μv), and the latency in milliseconds (ms) of the negative peak of the N100 VEP component will be analyzed separately for left and right-presented stimuli in correspondence of posterior electrodes [8]. Then, indexes of interhemispheric imbalance will be extracted. In particular, here we propose the Visuospatial Attention Bias Index (vABI), which is based on N100 peak amplitude and interhemispheric transmission time (IHTT) [29, 30], which is a further index of hemispheric imbalance, based on N100 peak latency.

### Sample size

The sample size calculation was based on the results and the population variance from previous studies, which employed the BIT as a primary outcome measure [16, 17, 28, 32]. In particular, we calculated the sample size on the BIT (primary endpoint) using the following formula:

$$ N=\frac{2{\left({z}_{\left(1-\alpha \right)}+{z}_{\left(1-\beta \right)}\right)}^2{\sigma}^2}{\delta^2} $$

where *N* is the final sample size; σ^2^: population variance established on previous studies [17] = 3.1; δ^2^: absolute error allowed for parameter estimation = 2.84; *z*: constant (corresponding to the value of the standardized normal random variable) that depends on the level of confidence desired for the estimation, fixing *α* = 0.05 and 1 − *β* = 0.80, (*Z*_1 − *α*_ + *Z*_1 − *β*_)^2^ = 10.5. The sample size resulting from the formula is 25.2. Consequently, the minimum sufficient simple to reach our primary outcome was established as 56 subjects (28 per group), to be enrolled over 2 years, assuming a 10% loss at follow-up.

### Safety assessment

The stimulation protocol (i.e., the intervention) was devised accordingly to the guidelines for stimulation by Müri et al. 2013 [33]:

1. The application should be easy to implement without neuroimaging and neuronavigation systems for the localization of the target area. Many studies, as an alternative to neuronavigation, adopt the international 10/20 localization system.
2. The total application time of the daily rehabilitation paradigm should not exceed ten sessions for 2 weeks. Indeed, protocols that provide everyday applications for more than 2 weeks are difficult to implement in rehabilitation centers and may not be tolerated by patients.

Indeed, when the r-TMS is administered according to the published international guidelines for brain stimulation protocols [33], it is a safe technique. The stimulation paradigm [17, 20] we will use in this study follows these guidelines. However, we will address any possible adverse events reported in the literature, as follows:

1. Mild headache after the session, usually not requiring any treatment (rather frequent). However, if requested by the patient, analgesics will be administered.
2. Some local annoyance in the stimulated area (frequent): this effect rarely requires the suspension of r-TMS, but should the discomfort reported to be excessive, the session will be interrupted.
3. Temporary loss of hearing (rare) for the duration of the stimulation session. In such a case, the session will be interrupted.
4. Epileptic crises (actually rather rare) may occur in predisposed individuals. To minimize this risk, subjects who have suffered from seizures during the acute phase or have a diagnosis of epilepsy will be excluded from the trial (exclusion criterion). Moreover, it is planned to have the EEG track recorded during the neurophysiologic pre-test assessment to exclude epileptiform anomalies. If epileptiform abnormalities are present, the participant will be excluded from the trial. Finally, should a convulsive episode occurs during the 15 days of treatment with r-TMS despite the above precautions, the latter will be immediately suspended, and the patient will be treated according to the standard hospital protocols for epileptic seizures.

The PI will report any adverse event in an ad hoc CRF. Should the treatment (either intervention or sham control) be suspended, the reason for the suspension will be reported in the CRF. Data on adverse events will be analyzed appropriately and included in the study’s final report.

### Assignment of interventions

#### Allocation

The PI will generate a blocked randomization list (2,2 per group) using the online software QuickCalcs (www.graphpad.com). Only the PI will have access to the randomization list after its generation.

#### Blinding

To ensure a double-blind assessment, pre-treatment assessments (T0) will be performed before randomization, whereas post-test (T1) and follow-up (T2) assessments will be carried out by an assessor who will not be aware of the randomization group. The visual scanning protocol will be administered by therapists unaware of the patient’s allocation to intervention or control arms. Patients themselves will be instructed not to reveal any information to the assessors on the brain stimulation treatment received.

To minimize biases deriving from inter-rater measurement errors, the following interventions will be performed before the start of the trial:

1. Collegial assessments of patients who are not candidates for enrolment by the various assessors involved in the trial to standardize the administration modalities and resolve any discrepancies between scoring procedures.
2. The subsequent development of an “assessment manual” containing all information for administering and scoring procedures.

### Data collection and management

All data will be anonymized, and a specific alpha-numeric code will be attributed to each subject after enrolment. All the assessors will be responsible for transfers the data they have collected on paper CRF into the corresponding electronic CRF in the study database shortly after their collection. The study database is configured so that only the PI will be aware of all patient details, including the randomization group, and this information will not be visible to any of the assessors. Statistical analyses over the complete dataset will be performed at the end of data collection. However, interim analyses approved by the local ethical committee may be performed.

### Statistical analysis

Changes in all endpoints will be assessed across groups (r-TMS plus VS and SHAM plus VS) and across time (at post-test and 12 weeks follow-up assessment). BIT, TAP, CBS, and the motor function tests (MI, TCT, and FIM^tm^) provide standard scores, separated for each test, and scores including a general performance with cutoffs that allow discriminating pathological performance. vABI and IHTT are continuous variables based on neurophysiological data. Differences in primary and secondary outcomes for each indicator will be analyzed between the pre-test baseline (T0), post-test(T1), and follow-up (T2) phases for both groups of patients (group r -TMS plus VS and SHAM plus VS group).

The pretest-posttest follow-up control group design is often analyzed with the posttest and follow-up as dependent variables and the pretest as covariate (ANCOVA) [34, 35] or with the difference between posttest and pretest as dependent variable (CHANGE) [36, 37]. The choice of one of these two methods should consider two assumptions: homogeneity vs. heterogeneity of the study population (i.e., group differences at pretest) and normal data distribution.

In general, ANCOVA is preferable in randomized controlled trials as treatment assignment is based on randomization [34–36], and this may prevent data heterogeneity. However, in heterogeneous data, a measure of gain is preferable (i.e., CHANGE). Both methods have more power if data are normally distributed [36].

In our study, we will verify first the two assumptions mentioned above for each outcome measure and, afterward, we will adopt the appropriate approach:

1. Homogeneity vs. heterogeneity: For each outcome measure, the ANCOVA assumption of homogeneity of regression slopes will be verified [38]. In case the assumption is violated, a CHANGE measure (i.e., a score of gain in the specific outcome) will be considered in a 2 × 2 mixed-model ANOVA with the between factor group and the within factor time (posttest and follow-up).
2. Normal distribution: Our primary outcome (the Behavioral Inattention Test) data will be analyzed after linear logistic transformation accordingly to the Rasch calibration, which ensures normal data distribution [39]. For the secondary outcomes, in the case normal distribution is not verified, a linear logistic transformation will be performed.
3. In the case assumptions are verified, a mixed-model ANCOVA will be used with a 2 × 3 design, where the “between” factor will be the randomization group and the “within” factor will be the time of assessment, with the covariate pre-test baseline (T0) used to control for the LHN level before the beginning of the rehabilitation protocols.

In all cases, a two-tailed *t* test for independent samples will be employed to investigate the differences between groups. Data analysis will be performed using the MatLab (The Mathworks Inc.) and SPSS (version 13) softwares. We will accept a significance level of 5% (i.e., *p* value = .05) corrected for multiple comparisons when needed. Data from all randomized patients will be included in the analyses. Should data be missing at follow-up, data will be analyzed according to the intention to treat principle. However, participants will be contacted by telephone in the proximity of follow-up to ensure participant retention.

### Oversight and monitoring

A dedicated member of the investigator team other than the PI will assess the completeness of data collection and be involved in monitoring all the clinical trial’s organizational, ethical, and scientific aspects. The PI will declare the end of the enrolment.

### Dissemination plans

All data will be treated so that the participants’ identities will be kept anonymous. Before publication, the trial results will be discussed within the research group, and authorship eligibility will be agreed upon with all contributors before publication. Professional writers will not be involved in the dissemination process. The trial data, suitably anonymized, will be made available upon request at the end of the trial.

### Ethical considerations

Treatments for LHN will be allowed after the post-test, should severe LHN-related symptoms persist. Furthermore, during the trial, the standard care treatments will be guaranteed within the inpatient rehabilitation setting for all patients in the r-TMS intervention and SHAM control groups.

### Roles and responsibilities

Patients are being enrolled within two centers: the Neuro-rehabilitation Unit of IRCCS Istituto delle Scienze Neurologiche di Bologna (which is the coordinating center) and the Intensive Rehabilitation Unit of “Villa Bellombra Hospital” in Bologna. Both centers received the required ethical approvals and authorizations from the local ethics committee (CE/AVEC num. 0078006 and CE/AVEC num. 148456).

## Discussion

Within the SMART-ATLAS study, we propose a new combined protocol for the rehabilitation of LHN in right hemispheric stroke patients. In particular, it combines two potentially effective treatments for the rehabilitation of cognitive and behavioral symptoms of left hemispatial neglect to investigate the advantages of a combined protocol compared to a single intervention. We will also combine brain stimulation (inhibitory r-TMS on the left intact parietal cortex) with a cognitive treatment (visual scanning), which will follow the stimulation. 

Many published studies have highlighted the efficacy of the brain stimulation and cognitive treatments administered alone for the rehabilitation of LHN syndrome after stroke [23]. However, the level of scientific evidence for the efficacy of combined approaches (i.e., brain stimulation and cognitive treatments) is still low because of factors such as small sample size, methodological bias (lack of double-blind studies or follow-up assessments), and contradictory results. To control for these biases, we will apply a double-blind, SHAM-controlled design within the context of an RCT, in which assessors and patients are blinded to the type of stimulation used, reducing any source of potential bias. Moreover, our outcomes consider several construct domains related to LHN. Indeed, we consider cognitive and behavioral aspects of LHN, together with disabilities in the motor domain and the activity of daily living. Importantly, we introduce indexes based on neurophysiological data to monitor changes induced by the treatment at a neurophysiological level. Thus, our design can provide a valuable comparison between the combined approach and the cognitive treatment alone, thus evidencing specific brain stimulation effects and contributing to identifying the most beneficial rehabilitation approach for LHN patients.

This study’s central hypothesis is that clinical symptoms of LHN are correlated to an imbalance in interhemispheric activity due to hyperactivity of the left hemisphere [1, 10–12]. Inhibitory r-TMS on the intact left parietal cortex reduces the left hemisphere activation and can rebalance interhemispheric activity. Therefore, this RCT will provide evidence of the clinical efficacy of the rebalancing effect of the r-TMS on a larger sample of stroke patients. In particular, we expect to observe better performances on clinical tests and batteries, indicating more substantial improvements in cognitive and behavioral symptoms of LHN in the r-TMS group compared to the control SHAM group. Furthermore, we expect that these more significant clinical improvements in the r-TMS group can also be reported in specific scales assessing motor independence in the activities of daily living. Finally, inhibitory r-TMS on the intact left parietal cortex could point out the particular effect of r-TMS protocols on neurophysiological correlates of LHN. More in detail, we expect to observe in the r-TMS group a more significant rebalancing effect than in the control group, as demonstrated by smaller amplitudes of vABI and earlier latencies of IHTTs at the post-test assessment. Finally, we expect to observe the persistence of this effect at follow-up.

Although both the visual scanning training and the r-TMS protocols alone have been demonstrated to be effective in the rehabilitation of clinical symptoms related to LHN, combinations of different therapies may boost the therapeutic effect. The rationale behind this hypothesis is that following a right hemisphere stroke, the neuronal loss may impair cognitive functions due to a functional deactivation of the related neuro-functional networks. Thus, the reactivation of those networks can support the rehabilitation of the compromised functions [40, 41]. Indeed r-TMS can lead to neuronal activity changes that outlast the stimulation itself and enable empowerment of a specific network or neuronal circuit [40]. This after-effect of the r-TMS can support cognitive task execution, thus facilitating experiential learning during cognitive training [40, 41]. In our study, inhibitory r-TMS is applied over the intact left parietal cortex; thus, the r-TMS modulation concerns the preserved areas and the interhemispheric connectivity [42–44]. As a consequence, r-TMS could increase the “responsiveness” of the peri-lesional areas and the interhemispheric connectivity [43] during a cognitive training protocol (i.e., the visual scanning), increasing its effectiveness compared to the SHAM condition, where only a placebo stimulation is applied before the training.

## Conclusions

The SMART-ATLAS protocol implements a novel therapeutic approach for the rehabilitation of attentive spatial deficits in stroke patients by combining brain stimulation with cognitive treatments. The proposed approach is easily applicable and relatively low-cost.

Although a TMS stimulator is necessary for trial implementation, the visual scanning protocol is very flexible in terms of materials and tasks, and mainly paper and pencil material is required. Moreover, visual scanning can be implemented at the bedside, and also patients with severe motor impairments can easily carry out the tasks.

Although this is a multicenter trial, only two centers are involved. Thus, the generalizability of results may be limited. Further studies and replications of our protocol can improve results generalization. Should protocol efficacy be demonstrated, it could be implemented in ordinary clinical practice, thus providing an additional therapeutic option to reduce the burden of LHN and improve the clinical outcome of patients with right hemispheric stroke.

### Trial status

The protocol here presented is the same registered on ClinicalTrials.gov (NCT04080999; title: “Repetitive Transcranial Magnetic Stimulation in Spatial Attention After Stroke”) on September 2019. Patient recruitment began on March 2019, and the estimated study completion date is March 2021. Fourteen patients have been screened, and ten patients were included in the study and randomized. Five of those patients have already completed the 12 weeks follow-up.

## Data Availability

Not applicable. No data is available at this point because the study is in process. The data will only be accessed at the end of the trial by the research team’s designated members.

## Abbreviations

BIT: Behavioral Inattention Test
CBS: Catherine Bergegò Scale
CCT: Conventional cognitive treatment
CT: Computerized tomography scan
EEG: Electroencephalography
EOG: Electrooculogram
ERP: Event-related potentials
FIM™: Functional Independence Measure
IHTT: Interhemispheric transmission time
LHN: Left hemispatial neglect
MI: Motricity Index
MRI: Magnetic resonance imaging
PI: Principal investigator
r-TMS: Repetitive transcranial magnetic stimulation
TAP: Test of Attentional Performance
TCT: Trunk Control Test
vABI: Visuospatial Attention Bias Index
VEP: Visual evoked potentials
VS: Visual scanning
